# Sustained Opening of the Blood-Brain Barrier with Progressive Accumulation of White Matter Hyperintensities Following Ischemic Stroke

**DOI:** 10.3390/brainsci9010016

**Published:** 2019-01-21

**Authors:** Imama Naqvi, Emi Hitomi, Richard Leigh

**Affiliations:** Neuro Vascular Brain Imaging Unit, National Institute of Neurological Disorders and Stroke, National Institutes of Health, Bethesda, MD 20892, USA; imama.naqvi@temple.edu (I.N.); emihitomi5@gmail.com (E.H.)

**Keywords:** white matter hyperintensities, blood-brain barrier, stroke, permeability imaging, cerebral small vessel disease

## Abstract

Objective: To report a patient in whom an acute ischemic stroke precipitated chronic blood-brain barrier (BBB) disruption and expansion of vascular white matter hyperintensities (WMH) into regions of normal appearing white matter (NAWM) during the following year. Background: WMH are a common finding in patients with vascular risk factors such as a history of stroke. The pathophysiology of WMH is not fully understood; however, there is growing evidence to suggest that the development of WMH may be preceded by the BBB disruption in the NAWM. Methods: We studied a patient enrolled in the National Institutes of Health Natural History of Stroke Study who was scanned with magnetic resonance imaging (MRI) after presenting to the emergency room with an acute stroke. After a treatment with IV tPA, she underwent further MRI scanning at 2 h, 24 h, 5 days, 30 days, 90 days, 6 months, and 1-year post stroke. BBB permeability images were generated from the perfusion weighted imaging (PWI) source images. MRIs from each time point were co-registered to track changes in BBB disruption and WMH over time. Results: An 84-year-old woman presented after acute onset right hemiparesis, right-sided numbness and aphasia with an initial NIHSS of 13. MRI showed diffusion restriction in the left frontal lobe and decreased blood flow on perfusion imaging. Fluid attenuated inversion recovery (FLAIR) imaging showed bilateral confluent WMH involving the deep white matter and periventricular regions. She was treated with IV tPA without complication and her NIHSS improved initially to 3 and ultimately to 0. Permeability maps identified multiple regions of chronic BBB disruption remote from the acute stroke, predominantly spanning the junction of WMH and NAWM. The severity of BBB disruption was greatest at 24 h after the stroke but persisted on subsequent MRI scans. Progression of WMH into NAWM over the year of observation was detected bilaterally but was most dramatic in the regions adjacent to the initial stroke. Conclusions: WMH-associated BBB disruption may be exacerbated by an acute stroke, even in the contralateral hemisphere, and can persist for months after the initial event. Transformation of NAWM to WMH may be evident in areas of BBB disruption within a year after the stroke. Further studies are needed to investigate the relationship between chronic BBB disruption and progressive WMH in patients with a history of cerebrovascular disease and the potential for acute stroke to trigger or exacerbate the process leading to the development of WMH.

## 1. Introduction

Abnormal magnetic resonance imaging (MRI) T2-signal change in subcortical white matter, often referred to as white matter hyperintensities (WMH), is a common finding in patients with a history of vascular risk factors [1] and has been associated with cognitive decline and dementia in elderly populations [2,3]. Comorbidity of WMH is frequently seen in patients presenting with an acute ischemic stroke. Following an ischemic stroke, the cognitive decline appears to accelerate [4]. Both acute stroke [5] and chronic WMH [6] have been associated with disruption of the blood-brain barrier (BBB). Diffuse disruption of the BBB in the white matter of patients suffering from an acute stroke has been identified [7]. Whether or not an acute focal event, such as a stroke, can trigger or exacerbate a remote effect on a chronic process, such as WMH progression, has not been established. Similarly, the role of BBB disruption in cognitive decline has not been determined. We present a case of a patient who, after suffering an acute stroke, demonstrated chronic disruption of the BBB and clear progression of WMH over a one-year period.

## 2. Materials and Methods

### 2.1. Study Details

Prior to any research activity, the patient described in this report was enrolled in our Institutional Review Board (IRB) approved National Institutes of Health (NIH) Natural History of Stroke (NHS) Study (identification number NCT00009243) which is an observational cohort study of stroke patients. MRI scanning was performed prior to treatment with IV tPA, 2 h after treatment, 24 h after treatment, 5 days after treatment, 30 days after treatment, 90 days after treatment, 6 months after treatment, and 1 year after treatment. Fluid attenuated inversion recovery (FLAIR) MRI was performed at all time points allowing for assessment of WMH. Perfusion weighted imaging (PWI), which can be post processed to produce BBB permeability images, was performed up to and including the 90-day scan but not on subsequent scans due to fluctuation in renal function. Thus, changes in BBB disruption were only studied during the first 90 days.

#### 2.1.1. MRI Scan Parameters

All MRI scans were acquired with the same MRI protocol on a 3T Siemens Skyra scanner (Siemens AG, Munich, Germany). Fluid attenuated inversion recovery (FLAIR) imaging parameters were: TR 9000 msec, TE 120 msec, 3.5 mm slice thickness, 40 slices. Blood-brain permeability imaging (BBPI) was derived from a dynamic susceptibility contrast (DSC) [5,8,9] acquisition with the parameters: TR 1200 msec, TE 25 msec, 7 mm slice thickness, 20 slices, 80 dynamics collected just prior to and during the injection of a weight-based dose of gadolinium (0.1mmol/kg of Gadobutrol (Gd-DO3A-butrol)) at a flow rate of 5 mL/s.

#### 2.1.2. White Matter Hyperintensity Assessment

WMH were assessed based on FLAIR imaging. WMH were segmented at each time point via a semi-automated method previously described [10]. In brief, the follow-up FLAIR images were co-registered to the initial baseline FLAIR image. WMH were defined as regions of interest 3.5 standard deviations above the average mean pixel intensity of normal brain tissue on each acquired FLAIR. Progression of WMH for the purpose of this study was based on comparing the WMH segmentation from the 3-month scan to the 1-year scan. This time frame was chosen to ensure that changes in the WMH were not attributable to the evolution of the acute ischemic stroke.

#### 2.1.3. Blood-Brain Permeability Image (BBPI) Assessment

BBPI were generated from the dynamic susceptibility contrast (DSC) images that were originally obtained for the purpose of perfusion weighted imaging (PWI). Post-processing of DSC PWI source images can be performed to generate BBPI as has been previously described [5,8,9]. BBPI images were co-registered to the FLAIR images so that a permeability overlay could be placed on the FLAIR images to demonstrate the spatial relationship between BBB disruption and WMH. BBPI expresses permeability as a percent leakage compared with the normal tissue. Leakage below 0.5% was considered as noise and removed from the image. BBPI signals that did not achieve a good fit during the BBB permeability modeling process (r^2^ < 0.9) were also considered as noise and were removed from the image. The end result is a color-coded BBB permeability heatmap.

## 3. Results

### 3.1. Clinical Case Description

**Figure 1:**
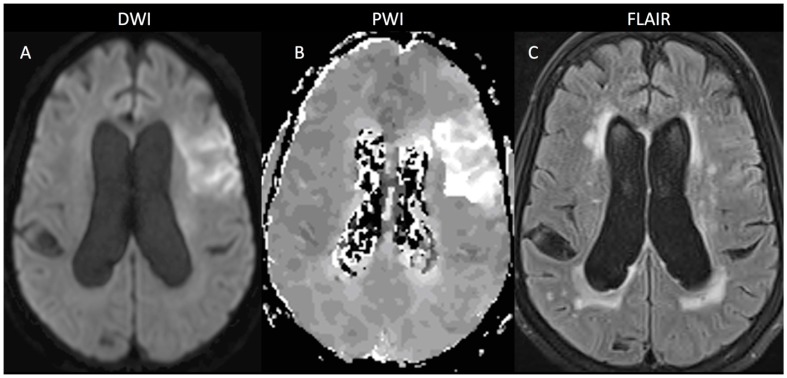
The MRI scan from the time of presentation (pre-treatment) is shown. Panel A is the diffusion weighted image (DWI) demonstrating an acute stroke in the left frontal lobe (radiologic convention). Panel B is a perfusion weighted image (PWI) showing the time-to-peak (TTP) map which demonstrates decreased blood flow in the region of acute ischemia. Panel C shows the FLAIR image which demonstrates that although the acute stroke has not yet developed increased T2 signal intensity, there are many areas of chronic white matter hyperintensity bilaterally.

An 84-year-old woman presented to the hospital emergency department after a sudden onset of right hemiparesis, right-sided numbness and aphasia upon waking; her NIHSS was 13. MRI evaluation showed diffusion restriction in the left frontal lobe (Figure 1A) with a corresponding area of decreased blood flow (Figure 1B) on perfusion weighted imaging (PWI). Gradient echo (GRE) imaging was negative for hemorrhage. There was minimal change on the FLAIR sequence in the region of the stroke (Figure 1C) despite bilateral WMH involving the deep white matter and periventricular regions. Although she had an unknown time of onset, she met the criteria for the MR WITNESS [11] clinical trial and received IV tPA. Two hours after treatment, her NIHSS had improved to 11, and by 24 h, her NIHSS was down to 5. At discharge, her NIHSS was 3; at 30 days after the stroke, it was 2, and by 90 days, it was 0 where it remained out to one year. Her modified Rankin score was a 1 at 90 days and at 6 months, however, it had increased to a 2 by the one-year time point.

#### 3.1.1. Imaging Findings

**Figure 2:**
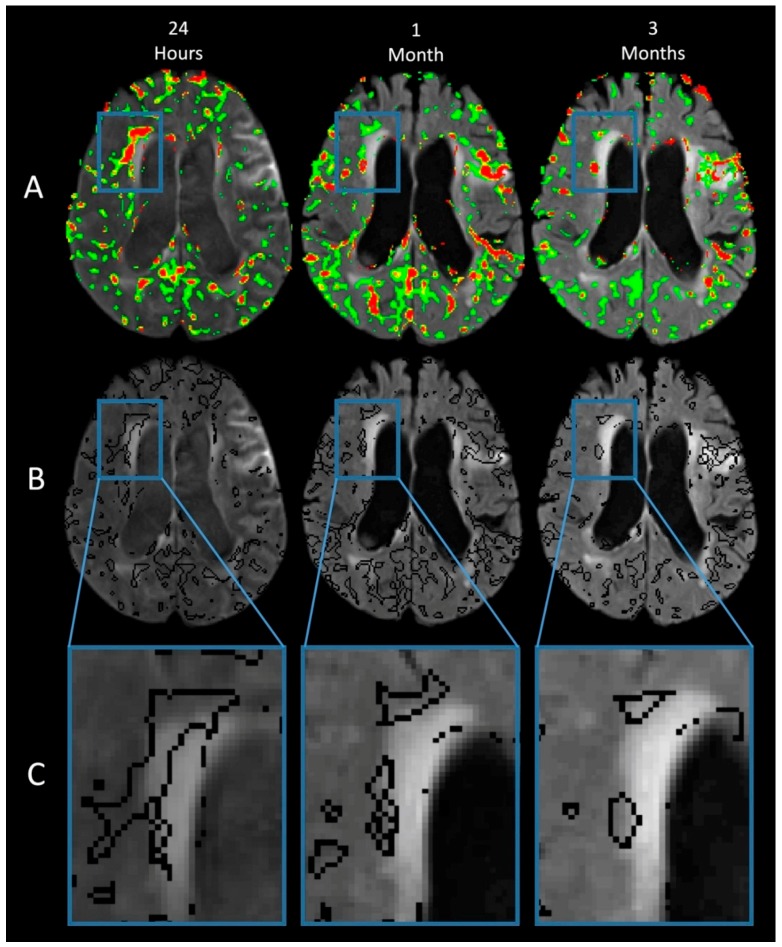
The same region of the brain is shown at three time points: column 1 is 24 h after the stroke, column 2 is 30 days after the stroke, and column 3 is 90 days after the stroke. Row A shows the BBB permeability heatmap superimposed on the FLAIR scan at each time point. The amount of BBB disruption is color coded: green 0.5% to 0.8%, yellow 0.8% to 0.9%, orange 0.9% to 1%, and red >1%. Although the BBB disruption has reversed in the area of the acute stroke (see text for description in results section), there is prominent BBB disruption in the contralateral hemisphere as pointed out in the boxed region. Note that the BBB disruption in the boxed area, as well as diffusely throughout the brain, is most severe immediately after the stroke and although it decreases over time, it persists for months. Row B shows the same BBB disruption from row A, except that now it is outlined instead of color coded. This allows visualization of the white matter hyperintensities (WMH) on the FLAIR below the BBB disruption. Row C shows the boxed region from row B magnified for examination of the WMH. Note the pattern of BBB disruption, often involving the normal appearing white matter (NAWM) adjacent to the WMH. BBB disruption involving the NAWM persists out to 90 days.

Figure 2 shows the same region of the brain at three time points: column 1 is 24 h after the stroke, column 2 is 30 days after the stroke, and column 3 is 90 days after the stroke. Row A shows the BBB permeability heatmap superimposed on the FLAIR scan at each time point. The amount of BBB disruption is color coded: green 0.5% to 0.8%, yellow 0.8% to 0.9%, orange 0.9% to 1%, and red >1%. The acute lesion appears bright on FLAIR at the 24-h time point in part due to gadolinium leakage into the CSF from an earlier MRI scan, however, the acute stroke does not demonstrate increased BBB disruption on the BBPI heat map at the time of the 24-h scan. This is consistent with our previous study demonstrating the reversal of BBB disruption after reperfusion in acute ischemic lesions [5] which may then be followed by a biphasic re-opening of the BBB within the ischemic lesion. However, in this case, areas remote from the acute stroke, particularly in the contralateral hemisphere, demonstrate widespread BBB disruption that is most severe at the 24-h time point but persists out to the 90-day time point.

Row B shows the same BBB disruption from row A, except that now it is outlined instead of color coded. This allows visualization of the WMH on the FLAIR image with the regions of BBB disruption superimposed. Row C shows the boxed region from row B, contralateral to the side of the infarct, magnified for examination of the WMH. Note the pattern of BBB disruption, often involving the normal appearing white matter (NAWM) adjacent to the WMH. BBB disruption involving the NAWM persists out to 90 days.

**Figure 3:**
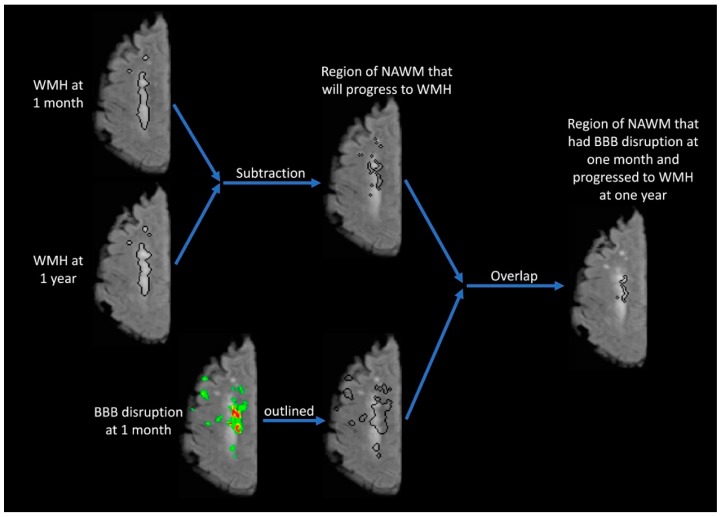
The purpose of this figure is to identify overlap between the location of BBB disruption 1 month after stroke and the locations of new white matter hyperintensities (WMH) at 1 year after stroke. The WMH region of interest (ROI) from 1 month is subtracted from the ROI at 1 year to identify regions of new WMH that are then superimposed on the 1-month FLAIR. Thus, the new ROI is an area of normal appearing white matter (NAWM) from the 1-month time point that will progress to WMH at the 1-year time point. Below that, the BBB disruption from the 1-month time point is outlined to form an ROI. The overlap between the BBB ROI and the ROI of new WMH is then identified to create a final ROI that identifies WHM progression that was preceded by BBB disruption.

The purpose of Figure 3 is to identify the overlap between the location of BBB disruption 1 month after stroke and the locations of new WMH 1 year after stroke. The WMH regions of interest (ROI) from 1 month and 1 year are compared to identify regions of new WMH which have developed over time. This ROI is then superimposed on the 1-month FLAIR image showing the area of NAWM that will progress to WMH. This ROI is then compared with the area of BBB disruption measured at 1 month. The lower images in Figure 3 outline the region of BBB disruption from the 1-month time point. This region of BBB disruption is then compared with the region of NAWM that will later progress to WMH to identify the overlap between these regions. The resulting image outlines the area of NAWM at the 1-month time point which has BBB disruption and will progress to WMH over the subsequent year.

**Figure 4:**
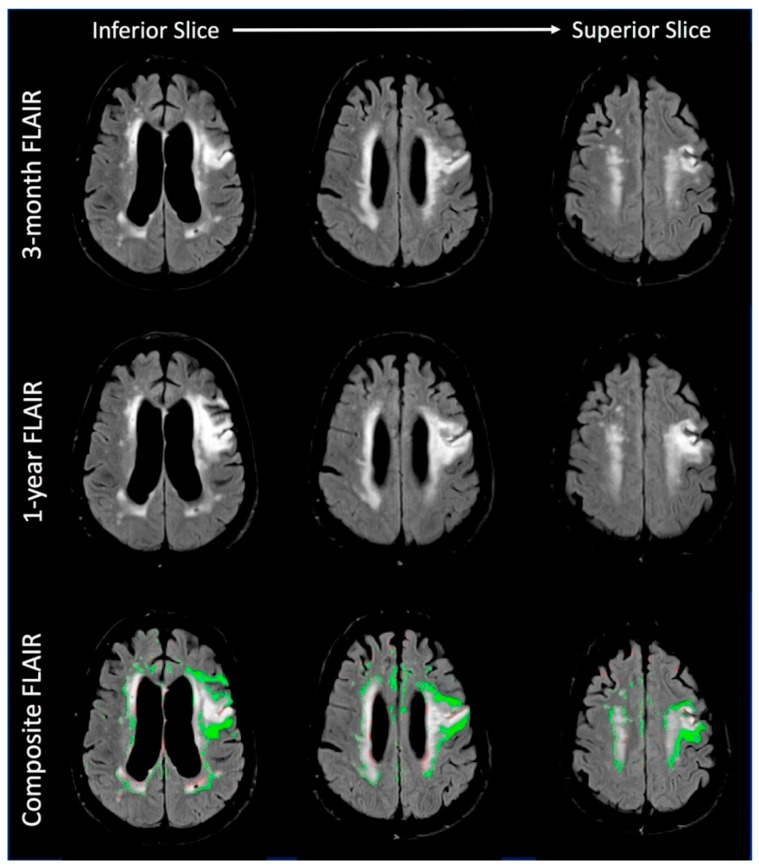
The increase in white matter hyperintensities (WMH) between the 3-month time point and the 1-year time point is shown for three regions in the brain. The first row is the FLAIR MRI from the 1-month time point. The second row is the FLAIR MRI from the 1-year time point. The third row is a composite image from the two time points with the increase in WMH represented in green. Red areas represent regions of decreased WMH and appear to mostly represent atrophy. The most dramatic region of WMH progression is seen in the areas immediately adjacent to the stroke.

Figure 4 shows the increase in white matter hyperintensities (WMH) between the 3-month time point and the 1-year time point for three regions in the brain. The 3-month scan was chosen in this case so that changes in the area of the acute stroke can be examined for chronic changes. The first row is the FLAIR MRI from the 3-month time point. The second row is the FLAIR MRI from the 1-year time point. The third row is a composite image from the two time points with the increase in WMH represented in green. Red areas represent regions of decreased WMH and appear to mostly represent atrophy. The most dramatic region of WMH progression is seen in the areas immediately adjacent to the region of the acute stroke. However, the bottom-right panel shows the progression of WMH in the contralateral hemisphere in the centrum semiovale as well. Thus, this figure demonstrates clear WMH progression over a 9-month period during a chronic phase of the cerebrovascular disease.

## 4. Discussion

By applying a novel technique for measuring BBB permeability on MRI to a single patient, we have been able to demonstrate an acutely elevated diffuse BBB disruption after a stroke that persisted for months and preceded the development of progressive WMH during the subsequent year. It is accepted that WMH can progress, and when it does, is associated with cognitive decline [12,13]. It is also now well established that the acute stroke itself can be associated with subsequent cognitive decline [4]. In our patient, BBB disruption appeared to be a possible link between the acute stroke and progressive WMH. Further studies will be needed to systematically test this proposal.

We have previously demonstrated that acute ischemia can have remote effects. For instance, patients suffering from an acute stroke demonstrate BBB disruption remote from the acute lesion that is proportional to their degree of cerebral small vessel disease [9]. Acute ischemia also appears to affect neighboring organs such as the blood-retina barrier in the eye [14]. In the current case, there is a suggestion that the focal acute ischemia is aggravating BBB disruption diffusely throughout the brain. If such a BBB diaschisis is occurring, it may explain why an acute focal event can have a global chronic effect.

We found a distinct pattern of BBB disruption that often straddled the border between WMH and the NAWM. This may be viewed as “the leading edge” of the process causing progressive WMH and thus is the most likely location for an active disease process. However, such a process would seem to be distinct from the accumulation of WMH that is due to multiple lacunar strokes. Previous studies of WMH-associated BBB disruption have also found the involvement of the NAWM [6,15]. Huisa et al. described a cohort of patients with WMH who had serial imaging of the BBB one year apart. They described WMH as an accumulating process and BBB disruption as a migrating process. In our patient, who was scanned serially over shorter time points, we found the BBB disruption severity to be dynamic but spatially stable. 

A recent consensus statement implicated BBB disruption as an early stage in the cascade of events that leads to progressive WMH [16]. Areas of the brain with reduced cerebrovascular autoregulation that are susceptible to intermittent hypoxia experience activation of the matrix metalloproteinases (MMPs) leading to degradation of the endothelial basal lamina and tight junction proteins that result in opening the BBB. This hypoxic pro-inflammatory environment leads to the accumulation of free radicals and proteases resulting in a non-immune mediated inflammatory demyelination [16]. This consensus statement concluded that WMH, which may have different pathophysiology than lacunar stroke, tends to enlarge over time “making it possible to determine a natural history that could potentially be altered by treatment.” [16] Prior studies have found that progression of WMH can be detected over a 3-year period whereas cognitive decline was detectable over a 6-year period [17]. From this, it seems possible that BBB disruption may be able to predict WMH progression, and thus cognitive decline, based on a single evaluation. Future studies should test this hypothesis and the potential role of acute stroke in triggering or exacerbating this process.

We did not perform cognitive testing with our patient, so we were not able to assess the effect of these MRI changes on cognition. However, the patient did experience a decrease in modified Rankin Scale at the 2-year time point suggesting a worsening in her functional status. Additionally, the NIH NHS study does not typically follow patients out to 1 year, so we are not able to comment on how common the findings seen in this patient are in the general stroke population. We are currently enrolling stroke patients in a study to undergo serial MRI and cognitive testing to investigate the link between BBB disruption and progressive WMH: https://clinicaltrials.gov/ct2/show/NCT03366129.

## 5. Conclusions

In conclusion, our case presents additional support for the hypothesized link between BBB disruption and progressive WMH. Additionally, our case suggests that BBB disruption may be a possible link between an acute stroke and subsequent vascular cognitive impairment.
